# Epidemiology and co-infection networks of pediatric respiratory pathogens in eastern China after COVID-19 restriction relaxation: a retrospective study

**DOI:** 10.3389/fcimb.2026.1795806

**Published:** 2026-04-13

**Authors:** Yanqun Sun, Wenshen Shao, Xu Wang, Ruizhe Yang, Wei Li, Changdi Xu, Ye Tian, Liming Cao, Man Tian

**Affiliations:** 1 Clinical Medical Research Center, Children’s Hospital of Nanjing Medical University, Nanjing, Jiangsu, China; 2 Department of Cardiothoracic Surgery, Children’s Hospital of Nanjing Medical University, Nanjing, Jiangsu, China; 3 Department of Public Health, Children’s Hospital of Nanjing Medical University, Nanjing, Jiangsu, China; 4 Department of Respiratory Medicine, Children’s Hospital of Nanjing Medical University, Nanjing, Jiangsu, China; 5 Department of Infectious Diseases, Children’s Hospital of Nanjing Medical University, Nanjing, Jiangsu, China

**Keywords:** co-infections, network analysis, pediatric epidemiology, respiratory pathogens, seasonality

## Abstract

**Objectives:**

To characterize the epidemiology, seasonal fluctuations, and co-infection networks of pediatric respiratory pathogens in eastern China following the relaxation of COVID-19 non-pharmaceutical interventions (NPIs).

**Methods:**

We conducted a retrospective analysis of 7,473 throat swab samples collected from children (≤18 years) presenting with acute respiratory tract infections (ARTIs) at a tertiary hospital in Nanjing between January 2024 and May 2025. Samples were tested for 13 common pathogens using a multiplex RT-PCR assay. Pathogen detection rates, temporal trends, and co-infection dynamics were analyzed using Kruskal-Wallis tests, time-series smoothing, and network analysis.

**Results:**

The overall pathogen detection rate was 61.8% (4,618/7,473), with no significant sex disparity (p = 0.267). Preschoolers (4–6 years) experienced the highest burden (68.3%), significantly exceeding other age groups (p < 0.008). Rhinovirus (RV, 16.9%), *Mycoplasma pneumoniae* (12.4%), and Respiratory Syncytial Virus (RSV, 9.0%) were the most prevalent pathogens. Distinct seasonal signatures were observed: RSV peaked in winter (25.3% in March 2025), *M. pneumoniae* dominated in mid-summer (22.8% in August 2024), and RV maintained high year-round activity. Co-infections occurred in 17.5% of positive samples, with M. pneumoniae-RV and Adenovirus-RV being the most frequent combinations. Network analysis identified RV and M. pneumoniae as central connectors within the intricate co-infection landscape.

**Conclusions:**

Pediatric respiratory pathogens have returned with strong, age-specific patterns in post-NPI eastern China. The pronounced “immunity debt” among preschoolers and the high rate of co-infections underscore the urgent need for enhanced multiplex surveillance and the expansion of vaccination programs for RSV and influenza.

## 1 Introduction

Acute respiratory tract infections (ARTIs) represent the foremost global cause of morbidity and mortality in the pediatric population, accounting for an estimated 37.8 million cases of lower respiratory infections and 502,000 deaths in 2021 in children under five years of age. Before the widespread implementation of COVID-19 non-pharmaceutical interventions (NPIs), temperate regions such as eastern China exhibited predictable, distinct seasonal patterns of respiratory pathogens. Respiratory Syncytial Virus (RSV) and influenza viruses typically drove winter outbreaks, Parainfluenza Virus (PIV) commonly circulated in spring, Rhinovirus (RV) maintained a year-round presence, and bacterial agents like *Mycoplasma pneumoniae* (*M. pneumoniae*) consistently peaked in late summer (Li et al., 2019; Moriyama et al., 2020). These inherent patterns are intricately modulated by environmental variables, including air humidity, temperature, and airborne pathogen survivability, as well as human behavioral factors such as school calendars and population density (Li et al., 2019; Baker et al., 2022). A critical complication in ARTI management is co-infection, defined as the simultaneous presence of two or more pathogens. Co-infections are often associated with significantly worse clinical outcomes, including protracted hospital stays, an increased requirement for oxygen support, and a higher propensity for unnecessary antibiotic prescription, thereby contributing to the global crisis of antimicrobial resistance (Asner et al., 2014; Deol and Miura, 2025; Vahlkvist et al., 2025).

The emergence of the COVID-19 pandemic and the resultant implementation of intensive NPIs—such as mandatory masking, social distancing, and nationwide school closures—dramatically curtailed the transmission of these common respiratory pathogens by severely limiting close interpersonal contact (He et al., 2023). Global surveillance data confirmed a profound suppression of these infections: the Southern Hemisphere reported a significant reduction in RSV detections during the 2020–2021 seasons, and influenza activity virtually disappeared (Di Mattia et al., 2021; Kim et al., 2021). Specifically in China, pediatric ARTI-related hospital visits declined, paralleled by a substantial decrease in test positivity rates for multiple pathogens (Ren et al., 2023; Wang and Shi, 2025). This period prompted expert concerns regarding the concept of “immunity debt”—a build-up of susceptible, unexposed children who, upon the relaxation of NPIs, would face the risk of unusually large and potentially severe outbreaks (Wang et al., 2024; Chen et al., 2025). This predicted scenario has since materialized globally: Australia experienced an unprecedented summer RSV surge in late 2020 to early 2021 that severely strained hospital capacity (Foley et al., 2021), Europe witnessed surge of pediatric respiratory tract infections after the COVID-19 pandemic (Lenglart et al., 2025), and cities across China reported a deluge of pediatric cases in late 2022, with emergency department visits escalating in major urban centers (Chen et al., 2025; Li et al., 2025).

Despite the clear epidemiological shift, current studies from China suffer from significant limitations, including small sample sizes (often fewer than 10,000 tests), restricted time frames (typically less than 18 months of post-NPI data), limited pathogen panels (often fewer than 10 agents tested), or a lack of detailed focus on co-infection dynamics (Zhou et al., 2025; Zhu et al., 2025). Regional variations were pronounced: in southern cities like Guangzhou, *M. pneumoniae* positivity surged from 1.2% to 29.4% post-NPI, displacing winter RSV dominance (Li et al., 2024). In northern regions, longitudinal records from Baoding city in Hebei province indicated respiratory co-infection rates rose from 25.1% in 2022 to 45.1% in 2023 among hospitalized children with community acquired pneumonia (Jiang et al., 2025). However, eastern China—home to major metropolitan hubs like Nanjing (with a population exceeding 9 million) and characterized by a distinct temperate monsoon climate—remains a significant knowledge gap. This region’s unique climatic and population dynamics result in patterns that differ fundamentally from the sustained humidity-driven trends of the south (e.g., Guangzhou’s *M. pneumoniae* summer shifts) or the dry, severe winter seasonality of the north (e.g., Baoding’s increasing co-infection). Our current study is specifically designed to address this critical regional void, particularly in the context of post-NPI pathogen resurgence, co-infection network architecture, and weekly temporal fluctuations. Bridging this gap is essential for developing targeted diagnostic strategies, optimizing vaccine distribution (such as for RSV immune prophylaxis), and enhancing syndromic forecasting during ongoing influenza-like illness seasons (Kim et al., 2017).

Utilizing retrospective data from 7,473 multiplex PCR-tested throat swab samples collected at Children’s Hospital of Nanjing Medical University between January 2024 and May 2025, the primary objectives of this study were to: (1) comprehensively describe pathogen detection rates stratified by age and demographic characteristics; (2) map detailed seasonal, monthly, and weekly trends, explicitly identifying evidence of post-NPI epidemiological rebound; and (3) elucidate the intricate co-infection landscape through network analysis and quantitative association measures to pinpoint the most common and clinically relevant pathogen pairs. This high-resolution analysis aims to provide a robust, evidence-based guide for localized public health interventions and contributes to the broader understanding of respiratory pathogen ecology in eastern China and similarly affected temperate climates.

## 2 Methods

### 2.1 Study design and setting

This investigation was a retrospective, observational study utilizing anonymized electronic medical records. The study setting was the Children’s Hospital of Nanjing Medical University, a 1,500-bed tertiary pediatric teaching hospital that serves as a referral center for Jiangsu, Anhui, and surrounding provinces, managing over 100,000 ARTI-related outpatient and emergency visits annually. We included all children aged 18 years and younger (≤18 years) who presented with typical ARTI symptoms (e.g., rhinorrhea, cough, fever exceeding 38 °C, tachypnea) and who underwent routine multiplex real-time PCR testing as part of their clinical management between January 1, 2024, and May 31, 2025. This cohort included both outpatient and inpatient cases. A consecutive case selection approach was employed to minimize selection bias. Exclusion criteria were incomplete patient records, primary complaints not related to the respiratory system, or duplicate samples from the same patient within a 7-day period. The study protocol was reviewed and approved by the hospital’s Institutional Review Board (IRB No. 2023-045). Given the retrospective, archival, and anonymized nature of the data, the requirement for individual patient consent was waived.

### 2.2 Specimen collection and pathogen testing

Throat swabs were collected by trained clinical staff using flocked swabs, rubbing the posterior pharyngeal wall and bilateral tonsillar areas several times while rotating to maximize contact, avoiding the tongue and oral mucosa. Swabs were immediately snapped into tubes with 3 mL sampling fluid (containing protein stabilizers, antibiotics, and buffer), oriented vertically, and transported at 2–8 °C for ≤4 hours to the central lab. Guardian consent was obtained for pediatric collections.

Pathogen detection used the 13 Respiratory Pathogen Multiplex Kit (Fluorescence PCR-Capillary Electrophoresis; Ningbo HEALTH Gene Technologies Co., Ltd.; Reg. No. 20183400518), per manufacturer instructions. Nucleic acids were extracted from swab suspensions via automated extractor (Smart LabAssist-16/32; same manufacturer) or manual kit, co-extracting 2 μL RT-PCR internal control (IC): all nasopharyngeal swab samples were processed in strict accordance with the laboratory’s standard operating procedure for this assay. Briefly, viral nucleic acids were extracted from 200 μL of sample supernatant using a magnetic bead-based nucleic acid extraction kit. The eluate was then subjected to a one-step reverse transcription and multiplex PCR to amplify the specific target genes of the 13 pathogens in a single tube. The resulting PCR amplicons were separated by size and identified using an automated capillary electrophoresis system. To ensure the accuracy and validity of the experimental results, a negative control, a positive control, and an internal standard were included in every batch of tests. Amplification (15 μL master mix + 5 μL eluate) occurred on a thermal cycler (e.g., Bio-Rad T100) with one-step RT-PCR: 50 °C/15 min (RT), 95 °C/2 min, touchdown (6 cycles: 94 °C/30 s, 65–60 °C/30 s, 72 °C/60 s), 29 cycles (94 °C/30 s, 60 °C/30 s, 72 °C/60 s), 72 °C/10 min. Products (1 μL) were separated by capillary electrophoresis on 3500xL Dx Genetic Analyzer (Thermo Fisher) with GeneScan 500 LIZ standard; peaks analyzed via GeneMapper (v1.5) (Chatterji and Pachter, 2006).

The assay detects 13 pathogens in one tube via amplicon sizes (107–313 bp): Influenza A (InfA; H1N1pdm09 via InfA+09H1, H3N2 via InfA+H3, others non-subtyped), Influenza B (InfB; Victoria/Yamagata non-subtyped), Adenovirus (ADV; B/C/E non-subtyped), Bocavirus (BoV), Rhinovirus (RV), Parainfluenza (PIV; 1–4 non-subtyped), Coronaviruses (CoV; 229E/OC43/NL63/HKU1 non-subtyped, excluding SARS-CoV-2), Metapneumovirus (hMPV; A/B non-subtyped), RSV (RSV; A/B non-subtyped), *Mycoplasma pneumoniae* (*M.pneumoniae*), Chlamydia (Chlamydia; C. pneumoniae/trachomatis non-subtyped). Positivity: peak height > PosS (high standard); < NegS (low standard) negative; gray zone repeat-tested (adjudicated positive if persistent). huRNA/huDNA/IC controls required > PosS for validity. SARS-CoV-2 excluded post-emergency. Limit of Detection (LOD): 0.098–0.4 Tissue Culture Infectious Dose (TCID) _50_/mL (viruses), 2,000–5,000 copies/mL (bacteria).

### 2.3 Data analysis

Demographic data were stratified by standard pediatric age categories: newborn (0–28 days), infant (29 days–1 year), toddler (1–3 years), preschool-age (4–6 years), school-age (7–12 years), and adolescent/teen (13–18 years), as well as by sex. The pathogen positivity rate was calculated as (Number of Positive Samples/Total Samples Tested) ×100. Temporal trends were summarized using both calendar monthly and International Organization for Standardization (ISO) weekly aggregations. Co-infections were defined as the detection of two or more distinct pathogens in a single throat swab specimen. Statistical comparisons of categorical variables (e.g., sex or age group differences) were performed using chi-square tests (χ^2^). For non-normally distributed data, such as rate variations across age groups, the Kruskal-Wallis H test was applied. Given the multiple pairwise comparisons across the six age groups, a Bonferroni correction was applied to maintain an overall significance level of α=0.05 (adjusted p<0.008).For the co-infection network analysis, we employed the ‘igraph’ package in R 4.3.1. Undirected graphs were constructed where nodes represented individual pathogens (sized proportionally to their total detection degree) and edges represented a co-infection event (weighted by the absolute count of co-occurrences, with thickness proportional to the weight). The strength of association between any two pathogens was quantitatively measured using the phi coefficient (φ) for binary data:


$$\varphi =\frac{\left(ab-bc\right)}{\sqrt{\left[(a+b)(c+d)(a+c)(b+d\right)}}$$

where a is the count of samples positive for both pathogens, b is positive for the first only, c is positive for the second only, and d is negative for both. The phi coefficient ranges from -1 (perfect negative association) to +1 (perfect positive association), with 0 indicating no association, offering a robust measure of non-random co-occurrence (Ferrari et al., 2024).

## 3 Results

### 3.1 Demographic characteristics and overall detection rates

**Figure 1:**
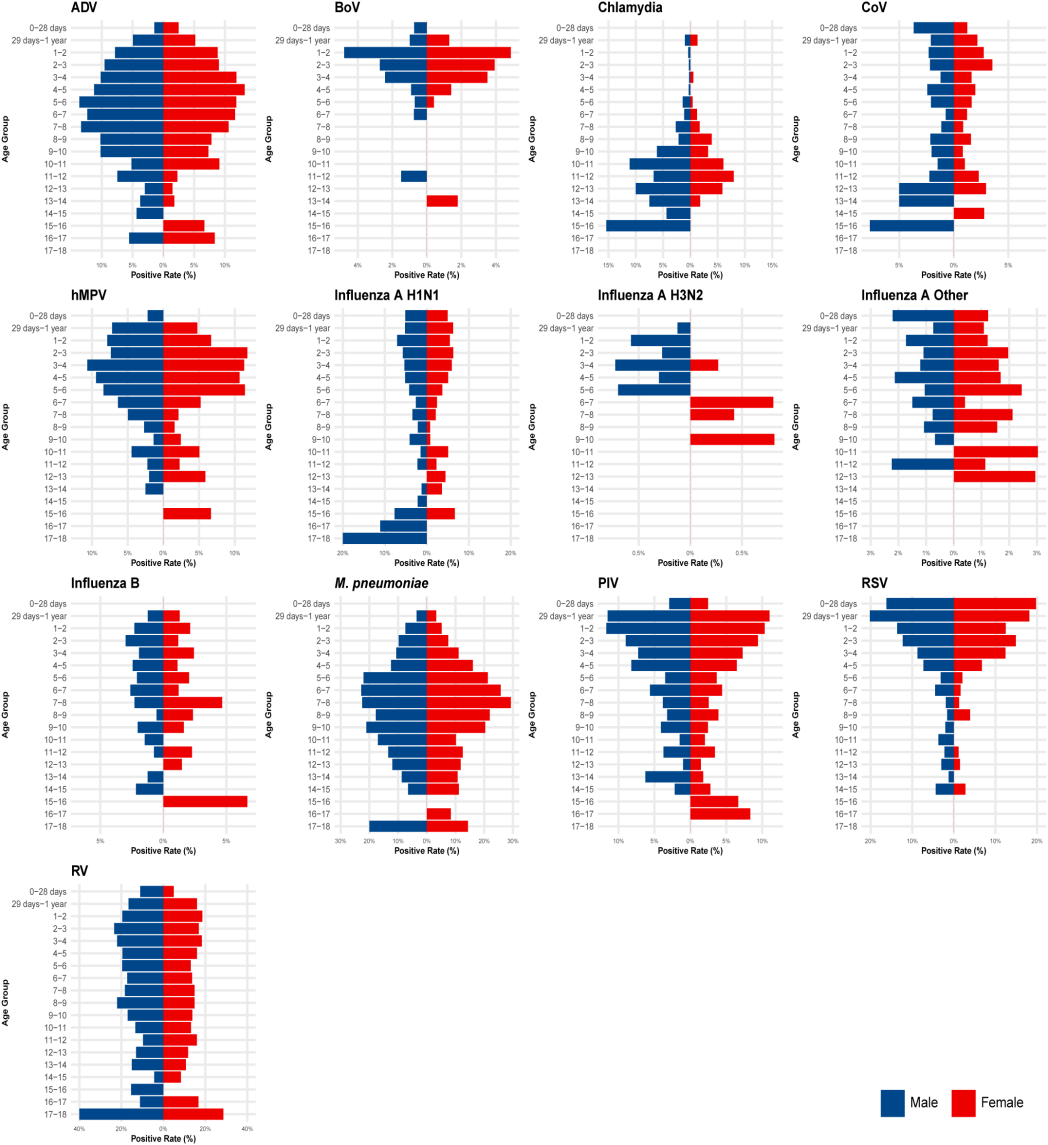
Age and sex characteristics of 13 respiratory pathogen infections in children. Multi-panel population pyramids: each panel for one pathogen (left-to-right: ADV to RV). X-axis: detection rates (%); negative = males (left bars), positive = females (right bars). Y-axis: age groups (0–18 years). Data peaks in 4–6 years (e.g., RV panel: 18–22% in 3–9 years).

A total of 7,473 throat swab samples were analyzed. Of these, 4,618 samples returned at least one positive result, yielding an overall pathogen detection rate of 61.8% (95% CI: 60.6%–62.9%) (**Table 1**). Males constituted a slightly higher proportion of the positive cases (62.6%, n=2,662), though this difference was not statistically significant (χ^2^ = 1.23, *df* = 1, *p* = 0.267). Significant differences in positivity rates were observed across the age groups (Kruskal-Wallis H = 456.7, *df* = 5, *p* < 0.001). The highest positivity rate was documented in preschool-age children (4–6 years), reaching 68.3%. Conversely, the lowest rates were observed in newborns (43.0%) and adolescents (38.8%). Bonferroni-adjusted pairwise comparisons confirmed that the positivity rate in the preschool cohort was significantly higher than in all other age groups (p<0.008 for all comparisons). Analysis of individual pathogens also revealed age-specific variations; for instance, RV rates peaked dramatically in preschoolers (22.04% in 4–6 years males) and showed a steep decline in older adolescents (14.84% in 13–18 years females) (**Figure 1**).

**Table 1:** Demographic characteristics and detection rates of 13 respiratory pathogens.

Characteristic	Counts			Positive rate (%)	χ²	*P*-value
	N	Negative, n	Positive, n			
Total	7473	2855	4618	61.8		
Sex					2.55	0.110
Female	3219	1263	1956	60.8		
Male	4254	1592	2662	62.6		
Patient Source					63.28	<0.001
Outpatient	5440	2227	3213	59.1		
Inpatient	2033	628	1405	69.1		
Age Group (years)					Fisher exact	<0.001
0–28 days	230	131	99	43		
29 days-1 year	1258	469	789	62.7		
1–3 years	1473	457	1016	69		
4–6 years	2000	634	1366	68.3		
7–12 years	2054	883	1171	57		
13–18 years	456	279	177	38.8		
Detection Season					34.98	<0.001
Autumn	1316	587	729	55.4		
Spring	1597	598	999	62.6		
Summer	1436	566	870	60.6		
Winter	3124	1104	2020	64.7		
Pathogen						
RV	7473	6209	1264	16.9		
*M. pneumoniae*	7473	6545	928	12.4		
RSV	7473	6797	676	9		
ADV	7473	6823	650	8.7		
PIV	7473	6959	514	6.9		
hMPV	7473	6978	495	6.6		
Influenza A H1N1	7473	7136	337	4.5		
CoV	7473	7325	148	2		
Influenza B	7473	7339	134	1.8		
Chlamydia	7473	7346	127	1.7		
BoV	7473	7358	115	1.5		
Influenza A Other	7473	7377	96	1.3		
Influenza A H3N2	7473	7457	16	0.2		

P values: chi-square test (or Fisher’s exact test when expected frequency <5) comparing positive rates across subgroups within each characteristic. Pathogen rows show individual detection rates against total samples (no between-group test). Excludes adults >18 years for Age Group. Seasons: Spring (Mar–May), Summer (Jun–Aug), Autumn (Sep–Nov), Winter (Dec–Feb).

### 3.2 Pathogen prevalence and breakdown

The positive detections were overwhelmingly dominated by viruses, which accounted for 92.4% of all positive results, compared to 7.6% for the two bacterial targets (**Table 1**). The top five most frequently detected pathogens were: RV at 16.9% (n=1,264), *M. pneumoniae* at 12.4% (n=928), RSV at 9.0% (n=676), ADV at 8.7% (n=650), and PIV at 6.9% (n=514). Other notable findings included hMPV at 6.6% (n=495) and total influenza (A and B subtypes) at 7.8% (Influenza A H1N1: 4.5% > Influenza B: 1.8% > Other A: 1.3% > A H3N2: 0.2%).

Grouping by pathogen family (**Figure 3A**, stacked bars) showed the dominance of Paramyxoviridae (RSV + PIV + hMPV) at 22.5%, Picornaviridae (RV) at 16.9%, and Mycoplasmataceae (*M. pneumoniae*) at 12.4%. A key finding regarding age distribution was the increasing prevalence of the bacterial pathogen: *M. pneumoniae* was substantially more common in school-age children (7–12 years: 15.2%) compared to near-zero prevalence in newborns (0%).

**Figure 2:**
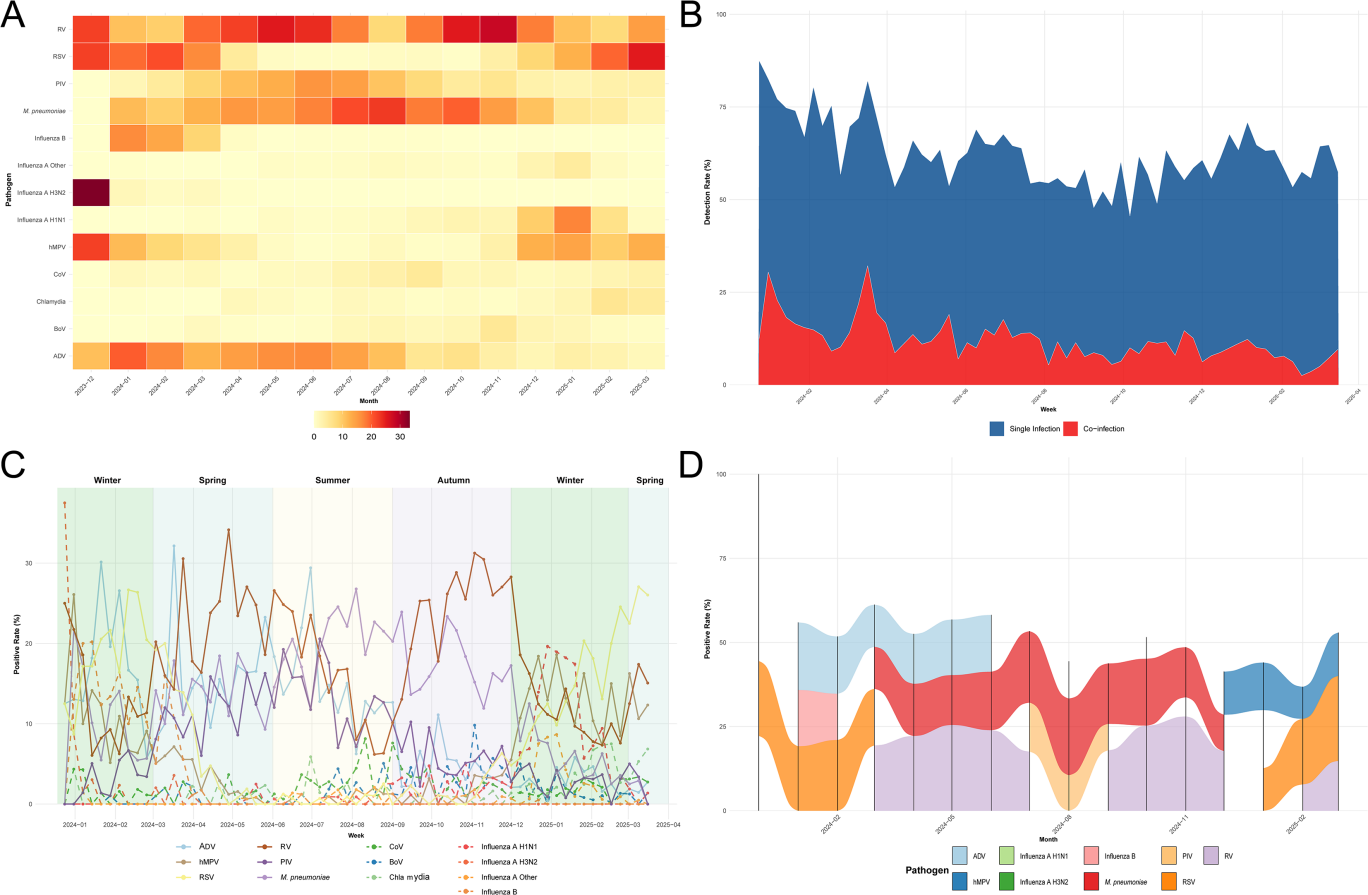
Seasonal and temporal trends of respiratory pathogens. **(A)** Monthly heatmap of positive rates (%); rows: pathogens, columns: months (Dec 2023–Mar 2025); color scale: low (blue) to high (red). **(B)** Weekly single (solid line) vs. co-infection (stacked bars) proportions (% of positives). **(C)** Weekly positive rates per pathogen (multi-lines; e.g., RV dashed). **(D)** Monthly top-3 ranked pathogens (bars, ranks labeled).

**Figure 3:**
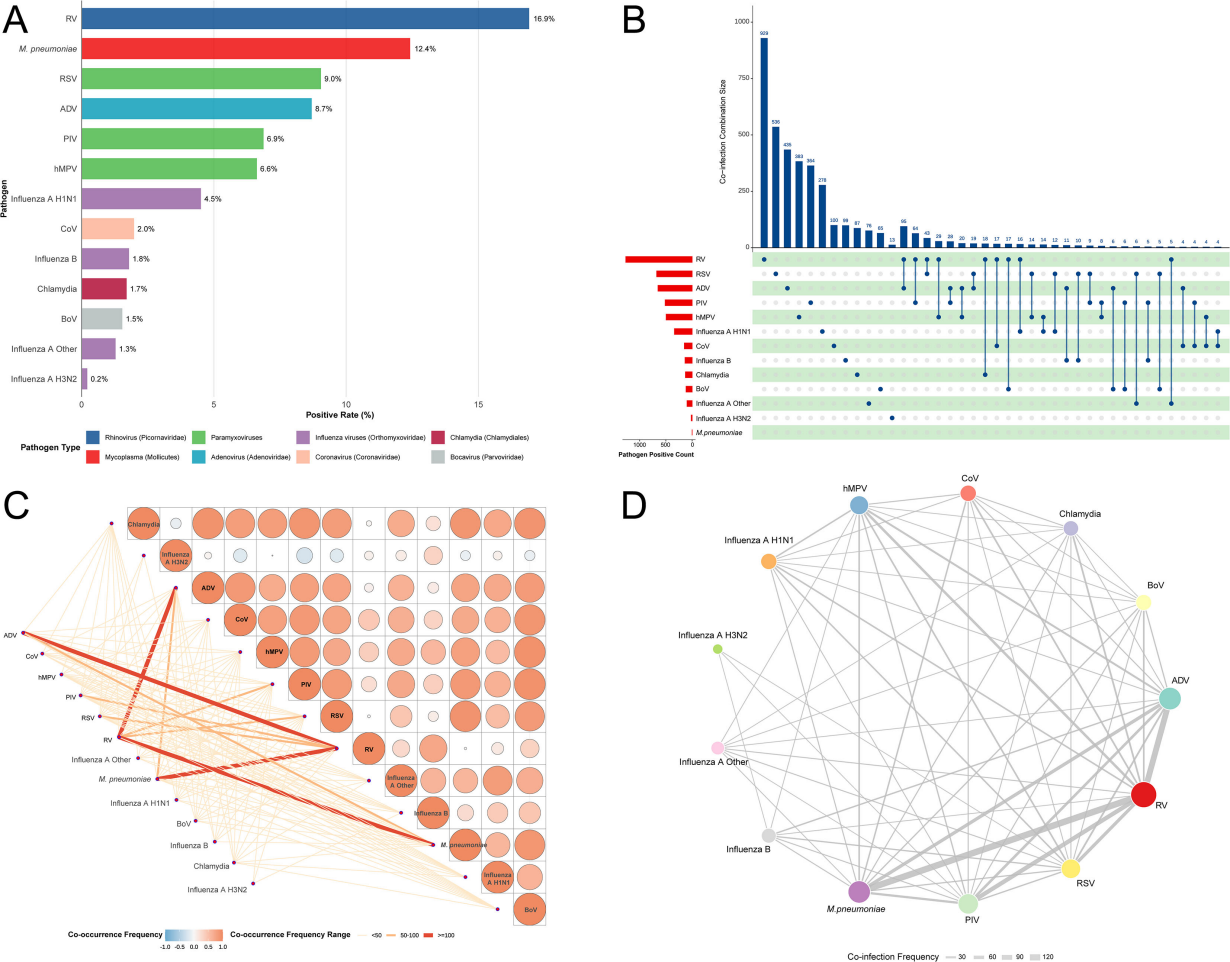
Co-occurrence and network of respiratory pathogens. **(A)** Bar plot of overall positive rates (%), grouped by family (e.g., Paramyxoviruses). **(B)** Top co-infection combinations (bar list) and binary detection matrix (rows: samples, columns: pathogens; 1=positive). **(C)** Co-occurrence heatmap; rows/columns: pathogen pairs; square size by frequency bin (<50 small, 50–99 medium, ≥100 large); color by phi index (-1 blue to +1 red). **(D)** Network graph; nodes sized by centrality (e.g., RV = 15.0), edges by strength (width, e.g., RV-*M. pneumoniae* = 5.0).

### 3.3 Seasonal and monthly trends

The monthly pathogen heatmap (**Figure 2A**) demonstrated a significant return to seasonality but with notable shifts compared to pre-pandemic patterns. RSV exhibited clear winter peaks, reaching 21.0% in February 2024 and an even higher 25.3% in March 2025 (ranking as the top-detected pathogen in both months). Influenza followed the winter pattern: Influenza B peaked at 16.7% in January 2024 (rank 3), and Influenza A H3N2 reached its highest rate at 33.3% in December 2023 (rank 1, though with low absolute numbers). hMPV and PIV showed a distinct spring-to-summer upward trend (hMPV: 11.6% in January 2024 to 12.8% in March 2025, with a peak of 22.2% in December 2023; PIV: 14.6% peak in July 2024), a change from their common past fall circulation.

Crucially, *M. pneumoniae* dominated the mid-summer months, peaking at 17.4% in June and 22.8% in August 2024 (ranking as the top pathogen in July and August). RV maintained a high baseline level year-round (10.9%–28.0%), securing the top rank in April–June and October–December 2024. Overall, the ranked bar plot (**Figure 2D**) confirmed that RSV/Influenza dominated winter (top 3 ranks in >80% of winter months), RV was the critical transitional agent (top 1–2 in approx60% of months), and *M. pneumoniae* ruled summer. The overall monthly positivity rate showed a stark 125% increase (4.0% in January 2024 to 9.0% in January 2025), with the rebound notably accelerating mid-2024 (July +150% rise).

### 3.4 Weekly temporal fluctuations

Analysis of weekly data (**Figure 2C**, overlapping lines) provided finer detail on the smaller wave dynamics. Notable weekly spikes included an ADV surge to 32.1% in mid-March 2024 (week of March 17), the RSV peak at 25.3% in early March 2025 (week of March 2), and a significant RV peak at 31.3% in early November 2024 (week of October 27). *M. pneumoniae* weekly highs demonstrated a possible link to socio-behavioral factors, such as the 21.2% peak during the week of August 4, 2024, coinciding with the end of summer holidays and preparation for school return. The overall positivity trend, calculated using a LOESS smoothing curve, showed an average increase of +0.12%/week, accelerating to +0.25%/week during the April–July 2024 period. The proportion of co-infections (**Figure 2B**) varied significantly across weeks (χ^2^ = 89.4, *df* = 51, *p* < 0.001), with the lowest single-infection rate at 45.6% (week February 25, 2024) and the highest co-infection rate peaking at 32.1% (week December 31, 2023).

### 3.5 Co-infection characteristics and networks

Co-infections were identified in 808 samples, representing 10.8% of all tested samples and 17.5% of all positive samples (**Table 2**), exceeding historical pre-pandemic norms. The average number of detected pathogens per co-infection case was 2.1. The dominant co-infection types were virus-virus (62.4%, n=504), followed by virus-bacteria (32.1%, n=259), with bacteria-bacteria being rare (5.5%, n=45). The frequency of co-infections exhibited a marked age-dependent increase (H = 234.5, *df* = 5, *p* < 0.001), rising steeply from 4.0% in newborns to a peak of 18.4% in preschoolers.

**Table 2:** Characteristics and co-infection proportions among positive respiratory specimens.

Characteristic	Counts			Co-infection proportion (%)	χ²	*P*-value
	N	Mono-infection, n	Co-infection, n			
Total Positive Samples	4618	3810	808	17.5		
Sex					1.83	0.177
Female	1956	1631	325	16.6		
Male	2662	2179	483	18.1		
Patient Source					1.23	0.268
Outpatient	3213	2664	549	17.1		
Inpatient	1405	1146	259	18.4		
Age Group (years)					Fisher exact	<0.001
0–28 days	99	95	4	4		
29 days-1 year	789	661	128	16.2		
1–3 years	1016	804	212	20.9		
4–6 years	1366	1115	251	18.4		
7–12 years	1171	977	194	16.6		
13–18 years	177	158	19	10.7		
Detection Season					7.66	0.054
Autumn	729	600	129	17.7		
Spring	999	808	191	19.1		
Summer	870	702	168	19.3		
Winter	2020	1700	320	15.8		

P values: chi-square test (or Fisher’s exact test when expected frequency <5) comparing co-infection proportions across subgroups within each characteristic. Excludes adults >18 years for Age Group. Seasons: Spring (Mar–May), Summer (Jun–Aug), Autumn (Sep–Nov), Winter (Dec–Feb).

The top five co-infection pairs (**Figure 3B**, list and matrix) were: *M. pneumoniae*-RV (110 cases, 13.6% of all co-infections), ADV-RV (92 cases, 11.4%), PIV-RV (62 cases, 7.7%), RSV-RV (42 cases, 5.2%), and ADV-*M. pneumoniae* (41 cases, 5.1%). Matrix sum totals confirmed the central role of RV and *M. pneumoniae*, implicated in 42% and 38% of all co-infection cases, respectively. The co-occurrence heatmap (**Figure 3C**) quantified the strength: the largest dark red squares (≥100 cases) confirmed RV as a central hub, notably with *M. pneumoniae* (122 co-occurrences). The overlaid phi coefficient identified strong positive associations (phi > 0.5) for several viral-viral pairs (e.g., RSV-hMPV 0.78) and notable negative associations (e.g., Influenza B-*M. pneumoniae* -0.25). The network visualization (**Figure 3D**) clearly positioned RV as the most central node (degree 12, size 15.0), strongly linked to both *M. pneumoniae* (edge weight 5.0, strength 122) and ADV (4.5, 110), establishing clusters dominated by viral agents (RV/ADV/PIV) and a significant mixed viral-bacterial cluster centered around *M. pneumoniae* and hMPV.

## 4 Discussion

This detailed retrospective analysis of 4,618 positive pediatric ARTI cases from a major tertiary center in Nanjing, eastern China, offers compelling evidence of a marked and heterogeneous resurgence of respiratory pathogens following the phased withdrawal of COVID-19 NPIs in late 2022. This surge aligns precisely with the hypothesis of “immunity debt,” where the population-level reduction in pathogen exposure during NPIs resulted in an accumulated reservoir of susceptible children, leading to amplified transmission upon the lifting of restrictions (Billard and Bont, 2023; Chen et al., 2025). A comprehensive multinational study across more than 25 European pediatric emergency departments confirmed this dose-response relationship: the greater the NPI-induced decline in respiratory tract infections (RTIs), the more aggressive the post-NPI increase, with some centers reporting bronchiolitis cases surging by up to 329% (Lenglart et al., 2025). In our specific eastern China cohort, this phenomenon manifested as a 125% escalation in monthly positivity rates from early 2024 to early 2025, with a distinct acceleration noted mid-year. This necessitates a critical re-evaluation of public health and hospital surge capacity planning for pediatric care, moving beyond simple historical averages.

RV and *M. pneumoniae* emerged as the two co-dominant pathogens, collectively accounting for nearly one-third of all positive detections (16.9% and 12.4%, respectively). RV’s persistent, perennial circulation, with peaks reaching 28.0% during transitional months, highlights its inherent environmental resilience. Similar post-COVID-19 surveillance in southern China (Shenzhen) and northern China (Shijiazhuang) also reported RV predominance, suggesting a national trend where RV now competes strongly with traditional winter viruses like RSV [21] (Zhao et al., 2024; Liu et al., 2025). The striking mid-summer dominance of *M. pneumoniae* (peaking at 22.8% in August 2024) is particularly noteworthy, echoing the unusual seasonal shifts observed in subtropical regions. This summer peak may be related to the timing of school and kindergarten re-openings or potentially exacerbated by the increasing prevalence of macrolide resistance, creating a unique regional risk factor (de Groot et al., 2025).

Conversely, RSV and influenza demonstrated only a partial restoration of their typical seasonality, often with significant delays. RSV’s winter peaks (e.g., 25.3% in March 2025) occurred 2–3 months later than the historical December onsets. Similarly, influenza subtypes (e.g., A H3N2 peak in December 2023) confirmed a winter presence but with asynchronous and variable rebounds. These disruptions mirror global observations where post-NPI RSV outbreaks initially exhibited year-round activity (e.g., China 2021-2022) before settling into delayed winter patterns (2023-2024). This is theorized to result from a combination of disrupted viral interference and the extensive immunity gap created by NPIs (Abu-Raya et al., 2023; Zhao et al., 2025). Influenza subtypes demonstrated only a partial restoration of their typical winter seasonality, often with asynchronous and variable rebounds. Such disruptions mirror global and Chinese observations of post-NPI influenza activity, where initial year-round circulation (e.g., 2021–2022) transitioned to delayed or extended winter patterns (2023–2024), driven by disrupted viral interference and an extensive immunity debt from NPI-reduced exposures (Xie et al., 2024).

The data revealed a disproportionate burden on the preschool-age cohort (4–6 years), which exhibited the highest positivity rate at 68.3%. This finding signals a critical vulnerability in children who were either born during the pandemic or experienced minimal pathogen exposure during their crucial early developmental years, vividly illustrating the age-specific toll of the immunity debt. Prior to the pandemic, peak detections were typically observed in infants (less than 1 year old). The post-NPI shift toward older preschoolers suggests a demographic where foregone early exposures are now compounded by high transmission rates in dense settings like kindergartens and daycare centers. This pattern aligns with national surveillance from Korea, where post-NPI RSV and influenza resurgences in the preschool age group strongly correlated with daycare attendance and a reduction in baseline population immunity, contributing to a 20%–30% increase in Pediatric Intensive Care Unit (PICU) admissions (Yoon et al., 2023). Our findings provide a strong justification for enhanced, age-targeted interventions, such as extending the use of RSV monoclonal antibodies (e.g., nirsevimab) and prioritizing annual influenza vaccination drives for the 3-6-year-old population (Billard and Bont, 2023).

A crucial finding was the high rate of co-infections, which affected 17.5% of all positive cases (10.8% overall), and peaking at 32.1% weekly. The dominant co-infections were primarily viral-bacterial dyads, with the *M. pneumoniae*-RV pair being the most frequent (13.6% of all co-infections). This highly RV-centric network (with RV involved in 42% of all co-infection events) suggests a potential synergistic mechanism of transmission, where the ubiquity and continuous circulation of RV may facilitate subsequent bacterial superinfection or compromise the host respiratory epithelium, making it more vulnerable to secondary agents (Bakaletz, 2017). Other Chinese data corroborate this finding: RV-*M. pneumoniae* co-infections comprised a substantial 30.8% of all mixed *M. pneumoniae* pneumonia (MPP) cases, and this co-occurrence was strongly associated with elevated macrolide resistance rates, increased odds of severe pneumonia, and prolonged hospitalization (Yuan et al., 2025). The age-escalating co-infection rates in our study (from 4% in newborns to 18.4% in preschoolers) suggest an inherent risk for heightened disease severity, as mixed etiologies are known to amplify inflammatory responses and increase the need for respiratory support (Chang et al., 2025). Future genomic and transcriptomic studies are needed to elucidate whether RV promotes *M. pneumoniae* adhesion or modulates immune evasion, which could inform the judicious use of adjunct therapies such as corticosteroids for refractory pneumonia cases (Papi et al., 2000).

Limitations must be considered when interpreting these findings. First, the single-center design, while ensuring consistent methodology and consecutive enrollment, limits the generalizability of the findings beyond urban Jiangsu province. Second, the use of throat swabs may have resulted in an under-detection of certain pathogens, particularly those with a higher yield in nasopharyngeal aspirates (Flynn et al., 2021). Third, the absence of clinical outcome data (e.g., Length of Stay [LOS], oxygen requirement) prevents a direct correlation between co-infection and severity, although the literature provides strong proxy evidence for this link. Fourth, the omission of SARS-CoV-2 testing based on post-emergency clinical protocols means that potential viral interference or co-circulation with this pathogen was not assessed, which may have impacted the seasonal dynamics of other viruses. In addition, SARS-CoV-2 was excluded from the analysis owing to methodological heterogeneity in detection assays, temporal misalignment of testing windows, and the study’s predefined focus on 13 common pediatric respiratory pathogens. Consequently, the generalization of our findings to clinical or epidemiological settings where SARS-CoV-2 is actively circulating may be limited. Finally, the study was limited to detection and did not include serological or genomic analyses, which would be essential for a deeper understanding of population immunity profiles and molecular-level resistance/transmission dynamics. Moving forward, multicenter, prospective studies that integrate comprehensive clinical outcomes, phylogenetics, and a comparison of rural-urban strata are imperative to fully characterize the respiratory epidemiology in the endemic era.

In conclusion, post-NPI eastern China has experienced a complex and heterogeneous respiratory pathogen resurgence characterized by delayed seasonality, non-classical summer dominance of *M. pneumoniae*, and a significantly amplified burden of co-infections, disproportionately affecting the preschool-age cohort due to immunity debt. Sustained, climate- and age-tailored surveillance programs, coupled with the routine use of multiplex diagnostics and expanded vaccination campaigns (for both RSV and influenza), are pivotal public health strategies required to effectively address this gap and fortify pediatric respiratory resilience in the enduring endemic landscape.

## Data Availability

The original contributions presented in the study are included in the article/supplementary material. Further inquiries can be directed to the corresponding authors.
